# Developmental Adaptation of Central Nervous System to Extremely High Acetylcholine Levels

**DOI:** 10.1371/journal.pone.0068265

**Published:** 2013-07-04

**Authors:** Vladimir Farar, Anna Hrabovska, Eric Krejci, Jaromir Myslivecek

**Affiliations:** 1 1st Faculty of Medicine, Institute of Physiology, Charles University, Prague, Czech Republic; 2 Centre d’Etude de la Sensorimotricité, Université Paris Descartes, CNRS UMR 8194, Paris, France; 3 Department of Pharmacology and Toxicology, Faculty of Pharmacy, Comenius University, Bratislava, Slovakia; University of Michigan, United States of America

## Abstract

Acetylcholinesterase (AChE) is a key enzyme in termination of fast cholinergic transmission. In brain, acetylcholine (ACh) is produced by cholinergic neurons and released in extracellular space where it is cleaved by AChE anchored by protein PRiMA. Recently, we showed that the lack of AChE in brain of PRiMA knock-out (KO) mouse increased ACh levels 200–300 times. The PRiMA KO mice adapt nearly completely by the reduction of muscarinic receptor (MR) density. Here we investigated changes in MR density, AChE, butyrylcholinesterase (BChE) activity in brain in order to determine developmental period responsible for such adaptation. Brains were studied at embryonal day 18.5 and postnatal days (pd) 0, 9, 30, 120, and 425. We found that the AChE activity in PRiMA KO mice remained very low at all studied ages while in wild type (WT) mice it gradually increased till pd120. BChE activity in WT mice gradually decreased until pd9 and then increased by pd120, it continually decreased in KO mice till pd30 and remained unchanged thereafter. MR number increased in WT mice till pd120 and then became stable. Similarly, MR increased in PRiMA KO mice till pd30 and then remained stable, but the maximal level reached is approximately 50% of WT mice. Therefore, we provide the evidence that adaptive changes in MR happen up to pd30. This is new phenomenon that could contribute to the explanation of survival and nearly unchanged phenotype of PRiMA KO mice.

## Introduction

The central cholinergic system belongs to one of the firstly appearing transmitter system in the brain and has been involved in important functions of central nervous system (CNS) such as cognitive processes [1], motor coordination [2], attention, circadian rhythms [3], food reinforcement and drug addiction [4] and synaptic plasticity [5].

Choline, transported from extracellular space by high affinity choline transporter (ChT), and acetyl coenzyme A (derived from Krebs cycle) form acetylcholine (ACh) [6] in the reaction catalyzed by choline acetyltransferase (ChAT) [7], [8]. Then ACh molecules are transported by vesicular acetylcholine transporter (VAChT) into synaptic vesicles [9].

The evoked release of ACh activates two classes of cholinergic receptors. Muscarinic receptors (MR, subtypes M_1_–M_5_) are G-protein coupled receptors [10]. Nicotinic receptors (NR) are pentameric ligand-gated ion channels of various subunit composition. Nine α subunits: α_1_–α_7_, α_9_, α_10_, four β subunits: β_1_–β_4_, and subunits γ, δ, ε were described [11]. In the CNS, the expression of α_2_–α_7_, and three β subunits: β_2_–β_4_ were described [12].

ACh action is terminated by acetylcholinesterase (AChE, E.C. 3.1.1.7). ACh can be also cleaved by butyrylcholinesterase (BChE, E.C. 3.1.1.8) [13]. In the CNS, these enzymes are mainly tethered to plasma membrane by anchoring protein PRiMA (**P**roline **Ri**ch **M**embrane **A**nchor) [14], [15].

All components of cholinergic system (CHT, VAChT, ChAT, NR, MR, AChE) appear already during the embryonal development in rodents and show a rapid age-related increase during the first three weeks of postnatal development. NR present subtype and brain region developmental differences [16]. In the rat brain, the activity of ChAT and protein level of VAChT increases slowly until postnatal day (pd)7 and then rapidly until pd24 [17]. A similar pattern has been shown for the development of cholinergic innervation in the cortex and hippocampus [18], [19].

In murine brain, AChE activity is measurable in embryonic day (E)9 and gradually increases 15-fold to E19 [20]. This increase continues after birth till the pd30 [20], [21]. The postnatal increase in AChE activity is also accompanied by increase in expression of PRiMA mRNA and protein [22]. In contrast to AChE, BChE shows only modest changes in activity during the postnatal development [23].

MR are transitorily expressed in the mouse blastoderm and in blastemic tissues during morphogenesis [24]. Between E17 and E18, MR are detectable in the brain, spinal cord and peripheral nerves [24]. After the birth, MR increase by pd25 of age and then become stable [25]. The number of NR in neonatal mouse brain is approximately twice the number in adult mouse brain [25] and the pattern of development differs from MR. There is an increase in NR level by pd10, followed by decrease by pd25 to the level that remain stable until adulthood [25].

*In vitro* and *in vivo* studies have demonstrated that in the adult rodent brain, the abundance and availability of MR at plasma membrane are controlled by the composition of neurochemical environment and depend on complex intraneuronal trafficking [26]. Overstimulation of MR, whether induced acutely or chronically (repeatedly), leads to the reversible decrease in the density of MR at plasma membrane [26].

Recently, we have found that in PRiMA KO mice, ACh levels are 200–300-times increased in the striatum [27], yet PRiMA KO mice are indistinguishable from (wild type) WT mice and present only marginal changes in behavior, motor skills and gait [27]. The central cholinergic system adapts to this huge increase in ACh by 20–60% decrease in MR level (accompanied by small, less than 20%, decrease in NR number). In the absence of PRiMA, AChE and also BChE, are not delivered to the plasma membrane but are retained in ER, the site of synthesis. Decrease in the number of total MR is similar in hippocampus, somatosensory cortex, motor cortex, olfactory tubercle, nucleus accumbens, and caudate putamen ranging between 37 to 63% reduction. This decrease is not a subtype specific, as we observed similar reduction in pirenzepine binding (usually considered as M_1_ MR, 27–51%) and AFDX-384 binding (usually considered as M_2_ MR, 33–67%). Although many transmitter systems (cholinergic muscarinic and nicotinic, GABAergic, dopaminergic, glutamatergic: AMPA, kainate, NMDA) and the ACh synthesis machinery were investigated [27], we only found changes in MR and NR levels. Despite low number, MR are still responsive to cholinergic stimulation as it can be deduced from retained thermoregulatory responses to oxotremorine (MR agonist) and also preserved locomotory responses to scopolamine (MR antagonist) in PRiMA KO mice, albeit the degree of responses is changed.

Therefore we suggest that the decrease in MR receptor level is the key adaptation to the lack of membrane anchored AChE and huge increase in ACh level in the brain of PRiMA KO mice. Here we determined when during the development this adaptation occurs. We tested hypothesis that the adaptation to gradually increasing ACh levels would start prenatally. This is important question at least for two reasons: 1) young animals are considered to be more sensitive to AChE inhibition than adults [28]; 2) a minimum of AChE activity necessary for survival [29] ranges between 17.5% (frontal cortex) and 92.1% (basal ganglia) while mice without PRiMA are indistinguishable from WT counterparts through visual inspection., i.e. without any sign of cholinergic hyperactivity with less than 5% of AChE activity. Also it is evident from mathematical model [30] that total AChE inhibition lead to LD_100_. That means that total inhibition of AChE (and also inhibition with residual AChE activity) is not reconcilable with life.

As a tool for testing our hypothesis, we employed radioligand binding studies together with measurement of AChE and BChE activity. Because of virtually identical changes in different MR subtypes and in different brain region we measured total MR in whole brain preparations.

## Methods

### Ethics Statement

All experiments were performed in accordance with the Czech Republic legislature and were approved by Animal protection Committee of the 1^st^ Faculty of Medicine, Charles University, Prague.

### Experimental Animals

Experiments were performed on WT and nullizygous mice for PRiMA (PRiMA KO) of both sexes at specific ages (E18.5, just after the birth (pd0), on pd9, pd30, pd120, and pd425). Concerning the first developmental points (the determination of embryonal day and pd0), we determined these using careful mice observation. Briefly, males were allowed to cover females for five hours in the morning and after 18 days in the evening the embryos were carefully removed from uterus immediately after decapitation of dams (E18.5), the brains were removed (with cerebellum, without adjacent parts), flash frozen in liquid nitrogen and stored at −80°C until membrane fractions were prepared [27].The newborn litters were decapitated and their brains removed just after birth (pd0). To obtain remaining age points, mice were decapitated at pd9, pd30, pd120 and pd425. The procedure of membrane preparation was the same as described before. Genotypes were determined by PCR with primers described elsewhere [15]. The genetic background of mice was a mixture equivalent to that for an F3 mating of B6D2 strain. Mice were maintained under controlled environmental conditions (12/12 light/dark cycle, 22±1°C, light on at 6 a.m.). Food and water were available *ad libitum*.

### Receptor Binding

We determined the total amount of MR in membrane preparations as described earlier [27]. The brains were homogenized with Ultra Turrax homogenizer by 3 pulses of 10 seconds in 15 volumes of cold 0.32 M sucrose. The homogenates were centrifugated at 4°C for 10 min at 1000 g to remove cell debris and nuclear fraction. The supernatant was removed and centrifugated for 55 min at 17 000 g to obtain membrane preparation. Supernatant was discarded and the pellet was washed once with cold 50 mM Na/K phosphate buffer ph 7.4, suspended in the same buffer and directly used for binding assay. The total amount of MR binding sites was determined in duplicates using 65–1600 pmol/l [^3^H]-QNB, non-specific binding was determined with 50 µmol/l atropine. The incubation was performed at 25°C and lasted 120 minutes as described previously [31]. The maximal amount of binding sites (B_max_) per mg protein (determined using BCA a method kit; Sigma) and the affinity constant (K_D_) was computed by non-linear regression using GraphPad Prism 5.01 program (GraphPad Software). Affinity constants (K_D_) were used for the “single-point” measurement in order to determine the total number of receptors while saving the amount of tissue, using saturating concentration of radioligand (2000 pmol/l [^3^H]QNB).

### Acetylcholinesterase and Butyrylcholinesterase Activity

Activity of AChE and BChE was determined by Ellman’s colorimetric method [32] modified for a 96-well microtiter plate reader (Tecan Sunrise) [33]. Briefly, the activity of AChE was assayed with 0.75 mM acetylthiocholine and 0.5 mM 5,5-dithiobis(2-nitrobenzoic acid) (DTNB) in 5 mM HEPES buffer pH 7.5.The total assay volume was 200 µl. 10 µg of membrane preparation was preincubated first with DTNB to saturate free sulfhydryl groups and with tetra(monoisopropyl)pyrophosphortetramide (iso-OMPA) (final concentration 0.1 mM) to block BChE activity during 30 min. The activity was measured at 412 nm. BChE activity was assayed as described for AChE except that butyrylthiocholine was used as substrate and AChE activity was blocked with 1,5-bis(4-allyldimethylammoniumphenyl) pentan-3-one dibromide (BW284C51) (final concentration 5 µM).

### Statistical Evaluation

Results are presented as mean±S.E.M. and each group represents an average of 3–9 animals. The binding data were evaluated using GraphPad Prism software (San Diego, USA). Statistical differences among groups were determined by two-way analysis of variance (ANOVA): for multiple comparisons an adjusted t-test modified SNK (Student-Newman-Keuls) correction was used.

## Results

### AChE Activity

**Figure 1:**
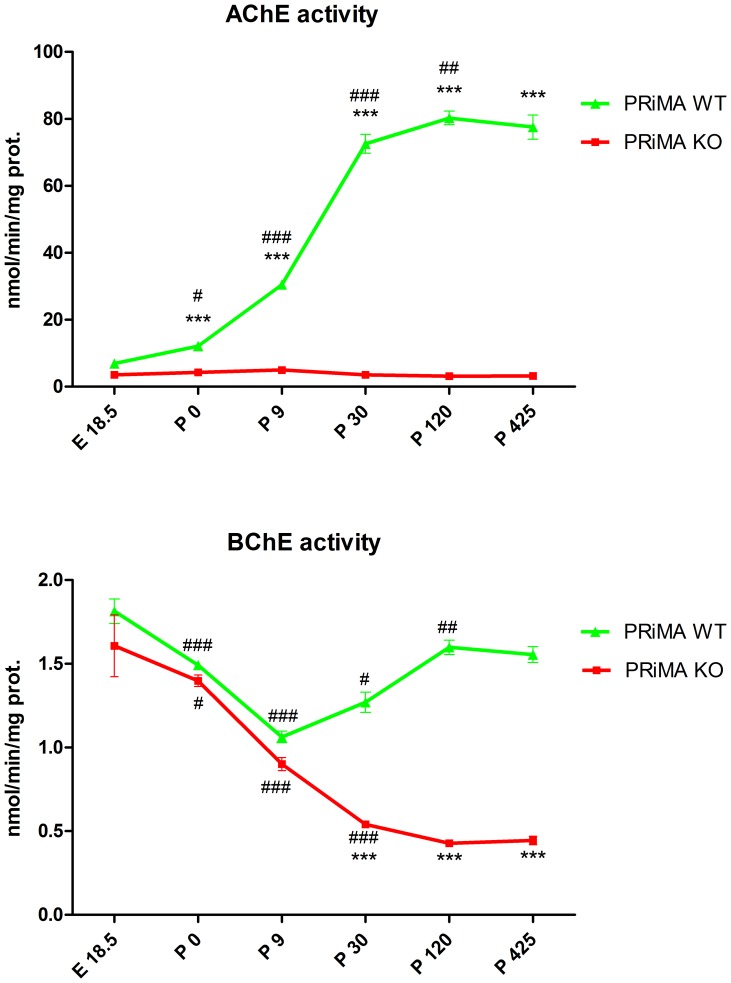
The development of acetylcholinesterase activity in the brain of PRiMA WT and PRiMA KO mice. **Bottom**: The development of butyrylcholinesterase activity in the brain of PRiMA WT and PRiMA KO mice. Abscissa: day of the development (E18.5, embryonal day 18.5; P0, postnatal day 0; P9, postnatal day 9; P30, postnatal day 30; P120, postnatal day 120; P425, postnatal day 425). Ordinate: the activity of acetylcholinesterase express as nmol/min/mg of protein. ***p<0.001, significantly different from PRiMA +/+. ^#^p<0.05, significantly different from previous day of development, ^##^p<0.01, significantly different from previous day of development. ^##^p<0.01, significantly different from previous day of development. ^###^p<0.001 significantly different from previous day of development.

The brain AChE activity did not differ between WT and PRiMA KO mice on E18.5 (see Figure 1). From the pd0 (zero), the AChE activity was significantly higher in WT animals than in PRiMA KO animals. AChE activity remained unchanged from the E18.5 till pd425 in PRiMA KO brain. In contrast, AChE activity steeply increased till pd30 of postnatal development, further increase occurred between pd30 and pd120 and the activity was stable up to the 1^st^ year of postnatal life in WT mice.

### BChE Activity

In the brain of WT mice, BChE activity decreased (see Figure 1) from prenatal period to pd9 then increased steeply until pd120 and remained stabilized thereafter. In PRiMA KO brain, BChE activity decreased drastically until the pd30 while it represented 42% of the WT value. It remained at the same value at all of following age points, but represented less than 30% of WT values.

### Receptor Binding

The affinity of receptors (K_D_) was not altered in membrane preparations of PRiMA KO mice (183.8±18.2 pmol/l vs. 143.2±27.6 pmol/l for WT and PRiMA KO mice, respectively) suggesting that we observed changes in receptor levels but not in their affinities to radioligand.

**Figure 2:**
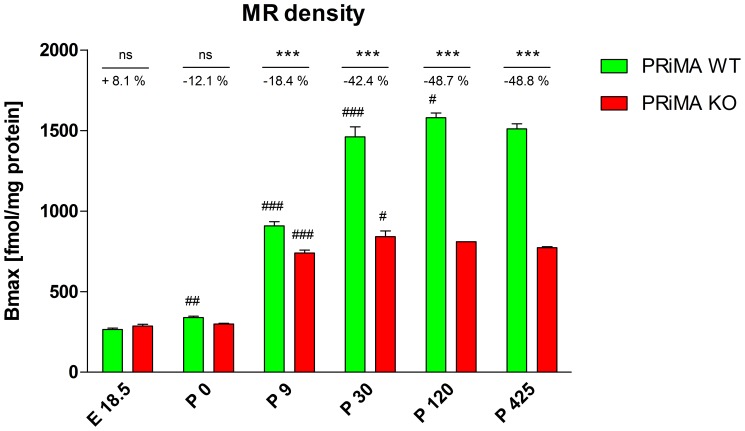
The development of number of binding sites in the brain of PRiMA WT and PRiMA KO mice. Abscissa: day of the development (E18.5, embryonal day 18.5; P0, postnatal day 0; P9, postnatal day 9; P30, postnatal day 30; P120, postnatal day 120; P425, postnatal day 425). Ordinate: the number of muscarinic receptor binding sites expressed as Bmax [fmol/mg protein]. ***p<0.001, significantly different from PRiMA +/+. ^#^p<0.05, significantly different from previous day of development, ^##^p<0.01, significantly different from previous day of development. ^###^p<0.001 significantly different from previous day of development.

The number of MR copied the development of AChE activity (see Figure 2). While the level of MR in WT brain increased steeply up to day pd30 and then less dramatically up to day pd120, in PRiMA KO brains, increase in the MR number stabilized at the level of pd30 (49% of WT value) and remained unchanged during further ageing.

## Discussion

Here we provide the data on gradual changes in MR level showing that the adaptation in brains of PRiMA KO mice is finished by pd30. This observation could contribute to the explanation of the viability and nearly unchanged phenotype of PRiMA KO mice [27]. Further it suggests how the organism could adapt to the conditions that are considered to be contradictory to the survival (like in AChE KO mice, [34]). Importantly, the AChE KO mice were indistinguishable from their littermates at birth by naked eye till pd7 when a clear difference was noticeable between AChE KO and WT mice [34] what supports the hypothesis about postnatal (and not prenatal) adaptation to increased ACh levels.

It is important to note that the development of AChE and MR in WT mice was similar as previously reported [16], [20], [24], [25] although we noticed subtle increase in AChE activity between pd30 and pd120 (9.7%).

BChE in brain is also associated with PRiMA [14]. Our data on postnatal BChE activity development in WT mice are similar to that previously published [23], [35]–[37]. BChE is expressed in white matter [38]. As the myelination starts during the postnatal development, decrease of BChE activity in PRiMA KO brain during an early development may be linked to the timing of myelin development. As far as we are aware, there is no other report on BChE prenatal development and in that view our data are new. Importantly, AChE and BChE (see Figure 1) activity developmental profile described here indicate that PRiMA becomes the main anchoring mechanism after birth.

**Figure 3:**
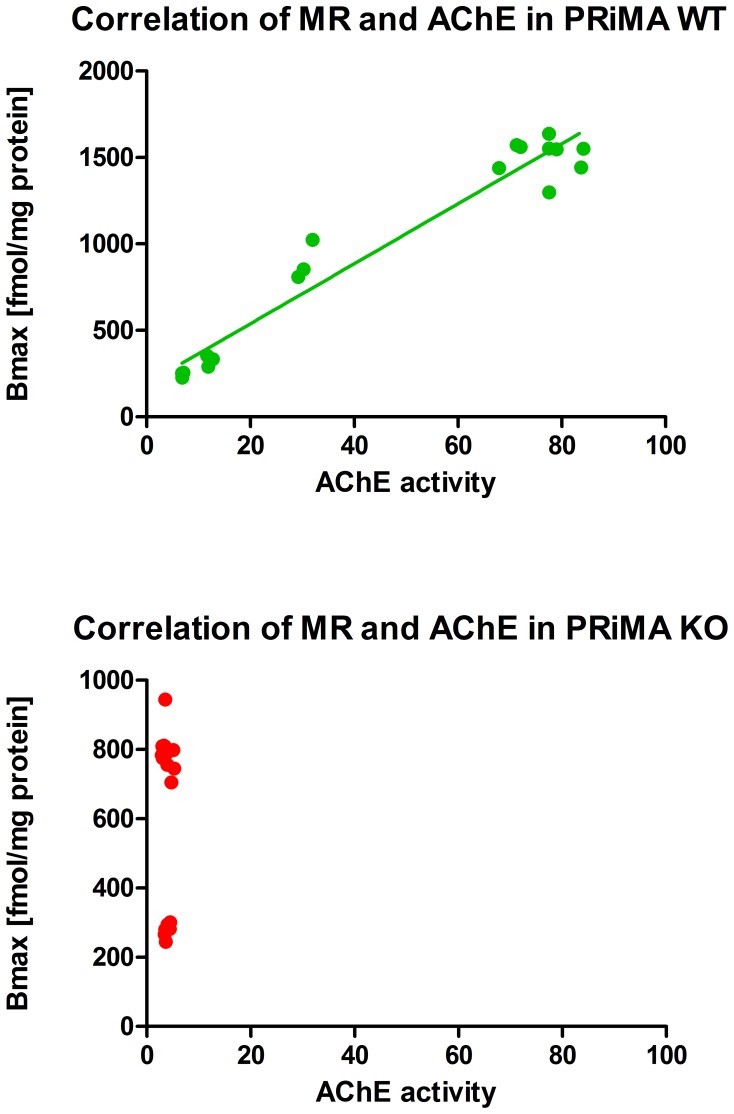
Correlation between AChE activity (abscissa) and MR density (ordinate). Top: Correlation in WT animals, bottom correlation in KO animals. In KO, there were no correlation, in WT we have able to discover significant correlation p<0.0001, Pearson r = 0.9727.

The increase in MR level slowed down from pd30 to pd120 (7.5% only). Therefore, it is possible to conclude that the development of AChE and MR is finished before pd30. Importantly, there is a clear correlation between AChE activity and MR density in WT mice (see Figure 3) which is not apparent in PRiMA KO mice due to almost null AChE activity in these mice.

As we have mentioned in Methods, we measured the total number of MR. Although five subtypes of MR exist, it is very difficult to characterize the changes of specific subtypes. The limitations are methodological. Muscarinic antagonist usually do not differ more than one order of magnitude [39] in pKi for M_1_–M_5_ subtypes and have similar pKi for two or more subtypes. Thus, it is possible to resolve it by multiple competition binding experiments but this makes the experiments extremely difficult. Another possibility how to determine MR subtype is determination using M_1_–M_5_ specific antibodies but validity of this methodological approach was challenged multiple times [40], [41]. Moreover, radioligand binding has also advantage that intact binding pocket (into G protein-coupled receptors transmembrane zones) is necessary for binding while antibodies can bind on cleaved receptor parts. As we have previously found similar changes in M_1_ and M_2_ MR here we demonstrate the changes in total number of MR what undoubtedly reflects the changes in specific subtypes.

In contrary to our hypothesis, the adaptation to extremely low AChE activity is not apparent during the prenatal period but the mechanisms occur during the postnatal development. Indeed, the MR density increases by 61% between pd9 and pd30 in WT mice while only by 13% in PRiMA KO. There are different hypotheses that may explain the adaptation delay of the MR density during the prenatal development. The production of ACh is still low and do not interfere with the regulation of MR localization and/or the development of blood-brain barrier is unfinished [42]. This would allow ACh to pass through blood-brain barrier into blood circulation where AChE activity is present [43]. Moreover, BChE activity is high in the serum, and could also contribute to ACh removal between E18.5 and pd30 before the blood-brain barrier is closed.

It is also possible to hypothesize that the degree of change in MR density reflects the excess of ACh. If the accumulation of ACh occurs then MR decrease should be gradual. But, as it is apparent form our data, MR do not change from day 30 to day 425 what suggest no accumulation of ACh after the pd30. Another explanation is increased MR synthesis and recycling in the presence of accumulated ACh.

Despite lower MR density in PRiMA KO mice after birth, the development pattern of MR in PRiMA KO mice is similar to that of WT mice. The age-related differences in total MR density in PRiMA KO mice are likely shaped by actual strengthening of cholinergic signaling. More generally, it can be suggested that gradual inhibition of AChE, such as treatment strategy in Alzheimer disease, or intoxication by AChE inhibitors present in environment, may be accompanied by gradual changes in MR abundance.

We can conclude that although it was shown multiple times that cholinergic system starts to develop in early prenatal stages, the development of cholinergic system in PRiMA KO mice is similar to that of WT mice. The differences in cholinergic system of PRiMA KO mice begin to be obvious after the birth in accordance with the maturation of the brain functions.
